# Neuralgia of the Teeth

**Published:** 1862-11

**Authors:** 


Neuralgia of the Teeth.—Odontalgia.—There is, perhaps, no form of disease of the nerves in any other part of the system, which is so common and has so little discrimination of condition, sex, or age, as what is commonly known as the *Toothache.* It is so very common that physicians are less frequently called upon for its alleviation than for any other pain causing near the amount of suffering which is located in any other part of the system. And the little success which attends ordinary attempts to alleviate the distress of an aching tooth would almost excuse the people from an application to a physician for relief.

Without any doubt, a part of this want of success in the attempt to alleviate the pain in this form of neuralgia arises from a want of sufficiently clear, distinct and definite knowledge, on the part of physicians, in regard to the nature of the disease, and, consequently, of a correct therapeutical application of remedies. There is no error so frequently committed, by some, as that of calling all painful affections of the teeth by the general and loose term of "toothache," under the impression that there is but one painful affection of the teeth, one specific disease, to be relieved by one remedy, or remedies of one character. I would as soon think of calling all forms of painful diseases of the head, scalp, skull, and brain simply "head-ache," and as requiring but one kind of treatment for their cure.

Pain in and about the teeth originates from a great variety of causes and conditions, most of them local, but some of them, doubtless, connected with the condition of the general system. Of the strictly local causes of toothache some are located at a distance from the seat of pain, and the pain will continue until the distant disease or derangement is cured. The connection of toothache with derangement of the pelvic organs of the female during menstruation or pregnancy, furnishes a familiar illustration of the pain originating in a locality far from the jaws.

The pain in a tooth is always quite annoying and sometimes excessively severe; and the final consequences of the disease which causes the distress, if allowed to progress unchecked, is often very unpleasant and life-long; and hence, the cause, the nature, and conditions, local or otherwise, which constitute the disease, in toothache, are worthy of very careful study and investigation.

Toothache may be the result of some derangement of the nerves, either of the tooth itself, of the face, or at some point at a distance from the tooth; of exposure of the nerve of the tooth from caries; of inflammation of the nerve within the tooth, without caries; of destruction of the neurilema, or the pulp of the nerve and lining of the tooth, with suppuration in the cavity of the tooth; of an inflammation of the membrane covering the fang of the tooth or lining the alveolar cavity; of rheumatism or gout, or an affection of the system *like* a slight attack of rheumatism; of inflammation of the tooth itself, as from a blow, or from a dental operation; or bone tumor on the tooth or the jaw, with pressure on the nerve; of fungus at the point of the fang; of collections of tartar around the fangs of the teeth; of exostosis.

*Toothache from Derangement of the Nerves.*—Among the common varieties of toothache, the most intractable and the most distressing is that which is the result of some derangement of the nervous system, local or general, and not of any disease of the tooth itself. Facial neuralgia is very frequently accompanied with a most distressing pain, which appears to have its seat in some particular tooth. Instances have occurred where nearly all the teeth on one side of the face, while still quite sound, have been extracted, one after the other, on the supposition that a tooth alone was the cause of the patient's suffering. So, also, of the toothache accompanying pregnancy. With some women pain in the teeth seems to be the inevitable accompaniment of the pregnant state.

Carious teeth may become very painful from neuralgia of the face or other parts of the system, or from the peculiar condition of the nerves attendant on pregnancy or derangement of the digestive or assimulative organs, but it is not seldom that teeth entirely free from caries become very painful from such causes. Whenever the teeth are already carious and become painful during pregnancy, or from derangement of chylopoïetic vicera, the caries seems to extend much more rapidly than it did before the tooth becomes painful. Persons of what is called *nervous* temperaments are quite liable to have an attack of toothache from any cause which produces nervous excitation ; and such persons, when the teeth commence to decay, find that caries is very rapid in its progress.

The pain of neuralgic toothache is usually quite acute, increasing in intensity to most acute agony, and then subsiding. It is often very irregular, darting and extending from tooth to tooth through the entire jaw. Often the pain remits, or even assumes a regular intermittent form, so marked as to be spoken of as the *periodic* toothache. But sometimes there is no absolute intermission of pain, and instead of the severe agony that has distracted the sufferer, the teeth will be acutely sensitive and tender, like those "set on edge" by an acid. This neuralgic tenderness is often made more apparent and more troublesome whenever the nerves of the ear are disagreeably excited, as from cutting the stem of an apple while paring it, or from the creaking of a door. When the teeth become tender from neuralgic pain or neuralgic excitation, any trivial operation on them, as from filing between them, becomes unbearable.

Neuralgic toothache, whether induced from the peculiar condition of other organs, as during menstruation or pregnancy, or idiopathic, is frequently difficult to diagnose, and almost always slow of cure. If the teeth are carious, perhaps they are more liable to suffer from simple neuralgic pains than sound ones are, but sound teeth do suffer excessively from this cause. If the tooth is quite sound, the pain is more likely to be paroxismal in character, without swelling or tenderness of the tooth or the gums, the pain extending along the jaw and involving more than one tooth, often attacking all on one side of the face.

*Treatment.*—For the cure of this variety of toothache, a very long list of remedies have been advised, and a great variety of applications, some of them quite mild and others *heroic* and dangerous. When this form of toothache is the result of the pregnant state, it will be apparent that no harsh or rash measures can possibly be justified. Nearly all writers on the diseases of females recognize the fact that pregnancy is not seldom followed by pain in the teeth, and they are quite emphatic in opposing *extraction* if the operation can be avoided. Death of the fœtus and abortion have followed extraction. But the pain of the tooth, also, if not relieved, may produce miscarriage, and hence the conscientious physician feels himself imperatively called upon to give relief. The ordinary "Toothache Drops" of the dentists will frequently afford, at least, a temporary respite from the pain. A very good combination is made as follows:

℞ Chloroform,

Camphor, āā ℥ss,

Alum (powdered), ʒj,

Morphia, sulph. ʒss. M.

or ℞ Chloroform,

Camphor, āā ℥ss,

Creosote, ʒss,

Oil of Pyrethrum, ʒj. M.

These preparations are very powerful, and must be used with caution; yet, in the hands of a physician they are safe. If there are any carious points in the teeth, the cavities should be carefully cleansed, and then cotton or lint, wet with a few drops of either of these mixtures, should be packed in the cavities and around the roots of the painful teeth, with dry cotton or lint between the wet lint and the cheek to protect the lining of the cheek from contact with the liquid.

Often the external application of these or any kind of toothache drops will not afford relief from neuralgic toothache. In such cases the gums may be carefully separated from the roots of the teeth, and an anodyne solution applied somewhat freely to the gums and the roots of the teeth. An injection of a solution of a salt of morphia into the gums has been found to give relief in some very severe forms of neuralgic toothache connected with pregnancy. The use of the scarificator and the syringe to bring the anodyne solution more distinctly in contact with the painful nerves, should never be forgotten.

If applications to the teeth and jaw do not afford relief, anæsthetics applied to the womb and the vagina may prove serviceable. A very convenient and safe method of applying a local anæsthetic to the mouth of the uterus, in this and other neuralgic affections, is to take a common bottle, fill it about half full with water in which is dissolved an ounce or so of the bicarbonate of soda. Perforate the cork and attach to it a flexible tube, as a large sized catheter. Then put into the bottle an ounce of tartaric acid, cork it, and let the gas which passes through the tube be directed into the vagina to the mouth of the uterus. Almost any patient can manage this herself when once told how.

*Toothache from exposure of the Nerve from Caries.*—Teeth may become carious, and even rot away entirely and not become painful. The pain of a carious tooth usually comes on quite suddenly as the result of some mechanical injury received by the nerve. A piece of food, while eating, the point of the tooth-pick, or the breaking of the tooth, has led to mechanical pressure on the nerve, causing sharp, quick, darting pain. This pain is relieved by almost any kind of stimulating applications, but returns again as soon as the air, or cold, or an acid is allowed to come in contact with the tooth. A wounded nerve in a carious tooth may remain simply *sensitive* for a long time, and then recover, or severe inflammation may follow, with suppuration and destruction of the tooth.

*Treatment.*—This form of toothache is nearly as common as the one already described, but not quite as difficult of cure, especially before acute inflammation sets in, after which, of course, another complication demands attention. If the loosened piece of the tooth is taken away from the nerve, all decayed or carious matter cautiously taken away, the cavity carefully sponged, and then filled with a pledget of cotton or lint wet with either of the toothache drops whose formulæ have already been given, or one into which the oil of cloves enters, and often renewed, in two or three days the nerve will be in a condition which will, perhaps, allow the dentist to cap over the nerve and plug the tooth with a temporary or even a permanent filling.

If the disease has already progressed so far that the nerve can not be restored to health, then the nerve must first be destroyed and the cavity plugged afterward.

*Inflammation of the Nerve without Caries.*—The causes which produce inflammation of the nerve of a tooth while the dentine is free from disease are not always known. The symptoms by which this condition can be determined are:—At first there is a disagreeable feeling of fullness, gnawing, and uneasiness, rather than marked pain. Mental preoccupation makes the patient lose consciousness of the suffering; but after a little time, as the inflammation becomes more developed, the pain increases, but the tooth does not become sensitive nor swollen. Cold air and cold water neither give relief nor aggravate the pain. After a little more time, cold does give relief to the suffering which has become somewhat severe and constant, if the tooth has not yet become tender on pressure.

*Treatment.*—Hydragogue, but not drastic, cathartics, anodynes, sudorifics, and local sialagogues, as the tincture of pyrethrum, with abstinence and rest, may suffice to relieve the patient when the inflammation extends no farther than the nerve. If, however, this disease is not arrested, it is quite sure to result in

*Destruction of the Nerve, the Neurilema, and lining of the Nerve Cavity, and Suppuration.*—Whenever the nerve of a tooth has become disorganized from inflammation, without or with caries of the tooth, and suppuration follows, if the tooth is not attended to, other forms of disease of the jaw and mouth may be produced. If a tooth becomes more than usually sensitive to heat and cold, feels *heavy*, tender, sore, and then painful, and afterwards a steady gnawing pain, with looseness, and slight apparent elongation of the tooth follows, it is probable that suppuration within the nerve cavity has already taken place. Inflammation of the nerve of a tooth, if allowed to progress, is quite sure to extend so as to involve the entire pulp, its covering, and the lining membrane of the cavity. When the pus forms, it may press out at the fang of the tooth and form an abscess in the alveola, or, by means of a sinus, escape into the soft tissues.

*Treatment.*—As soon as it is ascertained that there is pus within a tooth, the patient should at once be taken to a dentist, who will perforate the jaw and the tooth with a small drill, and let the pus escape. The point of selection where the orifice is to be made, and the size of the drill, will be decided by the dentist, who, by means of a small syringe, will wash out the cavity with such antiseptic and anodyne wash as the experience of that branch of the profession has taught him to rely on.

*Inflammation of the Membrane covering the Fang of the Tooth, and of the lining of the Alveolar Cavity.*—This is a common form of toothache, and attacks persons of all ages and both sexes. At times it prevails so extensively as almost to deserve to be called an epidemic. Sudden changes of the season, particularly from winter to spring, or the prevalence of sudden *colds* at any time, are often accompanied by an increase in the number of cases of this form of toothache. The pain, at first, is confined to the fangs of the teeth, which are tender, and all the old cases of caries begin to grumble and ache. The pain is dull, heavy, not very severe at first, but rather a soreness of the tooth and jaws; the gums become inflamed, swollen and tender, and of a dark color. Over the tooth which is most painful the gum is more swollen, puffy, and throbbing. The pain of the tooth, also, as pus forms, is throbbing and quite severe. The affected tooth becomes quite loose, and by the swelling of the inflamed membrane is pushed considerably above its neighbors. The face is not as apt to become swollen in this as in some other forms of toothache, although in all forms of the disease there may be pain in the head, loss of appetite, and inactivity of the digestive organs.

*Treatment.*—The pain may be relieved by free incisions in the gums and by cutting down as far as possible around the neck of the teeth into the alveolar cavities, and the application of anodyne washes. But this is the form of toothache where extraction of the tooth seems to be the only means of permanent relief.

*Inflammation of the Tooth.*—Quite often after an operation, as well as from other causes, the teeth become tender, sore, inflamed and painful. The only way such pain may be cured is to cure the inflammation. Tenderness of the teeth can often be relieved in a short time by an alkaline mouth wash. The soreness of the gums will usually yield to a wash of the tincture of horse-radish in which tannic acid has been dissolved. Chlorate of potassa in solution is an excellent wash for inflammation of the mouth and teeth. A wash made as follows is sometimes very good in this form of toothache:—

℞ Chloroform, ℨj,

Tannic acid, ℨj,

Creosote, gtt. xl. M.

This may be applied by means of lint to the gums and the teeth that are affected.

Usually, when the teeth have become inflamed because of an operation upon them, or from accidental injury, there is no demand for any but local treatment; but when the inflammation is the result of a cold, or rheumatism, or derangement of the digestive organs, the morbid condition of the general system will require its appropriate treatment in addition to the local treatment, before the disease can be subdued.

*Rheumatism.*—A chronic as well as acute taint of the general system with the poison which produces rheumatism or gout, is often the cause of a variety of toothache, characterized by a sense of soreness and tenderness rather than of acute pain, except there is also induced acute inflammation, when indications of inflammation of the covering of the fang of the tooth and of the lining of the alveolar cavity will also be present.

For the relief of the pain in the teeth the treatment already detailed may suffice. For the cure of the systemic derangement, the use of iodide of potassium, nitrate of potassa, tincture of prickly-ash, some preparation of phytolacca, and benzoic acid, may be demanded. The first-mentioned articles in those cases complicated with rheumatism, and the acid in cases complicated with the gouty diathesis. When toothache accompanies an acute attack of the gout, it is well to apply lint saturated with the tincture of colchicum almost constantly to the tooth and gums.

*Fungus at the point of the Fang.*—Sometimes teeth which are apparently sound are brought to the notice of physicians and dentists, which have been quite painful for a long time, and yet there have been no symptoms of acute inflammation of the tooth or its envelope, and no symptoms of the formation of pus. The tooth has been carious, perhaps filled, and the nerve dead. On extracting these teeth, there is observed at the point of the fang a soft, pulpy mass, which has been called a *fungus*. This mass may not be larger than a pin's head, but is often as large as a small pea, and is very fœtid. As active hemorrhage is quite apt to follow the extraction of teeth with fungus, it is probable that the so-called fungus is in reality the changed blood-vessels and the changed lining of the cavity of the tooth with, perhaps, the neurilema.

If a tooth has had its nerve destroyed and the cavity has become filled with a soft, deep red substance, which bleeds profusely if touched, has no pain of a darting or throbbing character, but the pain is steady, and the tooth is very fœtid, the disease is almost surely of the character here described.

*Treatment.*—When it is ascertained that a tooth has taken on this form of disease, it should at once be made to bleed profusely, and the gum should be separated from it and scarified. As soon as the pain is relieved, the fungus should be completely eradicated in the same manner as the nerves of teeth are destroyed, and then, if thought proper, the tooth may be filled. If the morbid growth can not be controlled, the tooth must be extracted.

*Collections of Tartar.*—Sometimes tartar collects in such quantities around the necks of the teeth as to loosen them in their sockets, and make them tender, sore and painful. If it is dark-colored, with a rough, uneven surface, quite hard, and difficult of removal, the indications are that the general health of the patient requires attention. Ordinarily, however, if the tartar is cautiously and skillfully removed, and the mouth and gums and teeth carefully washed after each meal with good soap and water, and then with the tincture of myrrh, the teeth will be restored to health in a short time.

*Exostosis.*—When there has been formed a bony tumor on the root of a tooth, which, by its mechanical pressure, causes toothache, extraction is the only cure. The existence of this disease can seldom be determined until the tooth is drawn.

Pain in a tooth, as pain in any other part, must be more or less directly connected with the condition of the nerves, as the nerves are the only part of the organism which are capable of conveying to the brain the sensation called pain. The common term, *toothache*, is so frequently applied to all forms of pain in the teeth, and is so easily repeated, that even physicians have not always had a clear idea of the *varieties* of pain in the teeth, and of the various causes of these various pains,—and hence it is necessary to enumerate some of them as has been done in the preceding pages.—*Jour. of Rational Medicine.*

